# 4-(2,6-Dibromo-4-fluoro­anilino)pent-3-en-2-one

**DOI:** 10.1107/S1600536811044606

**Published:** 2011-10-29

**Authors:** Gertruida J.S. Venter, Gideon Steyl, Andreas Roodt

**Affiliations:** a Department of Chemistry, University of the Free State, PO Box 339, Bloemfontein 9300, South Africa

## Abstract

The title enamino­ketone, C_11_H_10_Br_2_FNO, has a roughly planar pentenone chain; the maximum displacement of an atom from the pentenone plane is 0.071 (4) Å. The dihedral angle between the benzene ring and the pentenone unit is 77.2 (1)°. Inter­molecular C—H⋯Br and C—H⋯O inter­actions, as well as an intra­molecular N—H⋯O inter­action, are observed. In both methyl groups, each H atom is disordered equally over two sites.

## Related literature

For synthetic background, see: Shaheen *et al.* (2006 ▶); Venter *et al.* (2010*a* ▶,*b* ▶). For applications of enamino­ketones, see: Brink *et al.* (2010 ▶); Chen & Rhodes (1996 ▶); Nair *et al.* (2002 ▶); Otto *et al.* (1998 ▶); Pyżuk *et al.* (1993 ▶); Roodt & Steyn (2000 ▶); Steyn *et al.* (1992 ▶, 1997 ▶); Tan *et al.* (2008 ▶); Van Aswegen *et al.* (1991 ▶); Xia *et al.* (2008 ▶). For related ligand systems, see: Venter *et al.* (2009*a* ▶,*b* ▶).
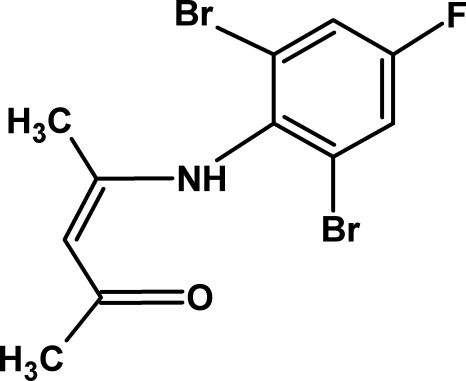


## Experimental

### 

#### Crystal data

C_11_H_10_Br_2_FNO
*M* *_r_* = 351.02
Orthorhombic, 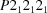

*a* = 8.7710 (3) Å
*b* = 10.8710 (4) Å
*c* = 12.6720 (4) Å
*V* = 1208.27 (7) Å^3^
*Z* = 4
Mo *K*α radiation
μ = 6.70 mm^−1^
*T* = 100 K
0.66 × 0.25 × 0.18 mm

#### Data collection

Bruker X8 APEXII 4K KappaCCD diffractometer
Absorption correction: multi-scan (*SADABS*; Bruker, 2004 ▶) *T*_min_ = 0.096, *T*_max_ = 0.379
20084 measured reflections
2624 independent reflections
2381 reflections with *I* > 2σ(*I*)
*R*_int_ = 0.084

#### Refinement

*R*[*F* ^2^ > 2σ(*F* ^2^)] = 0.028
*wR*(*F* ^2^) = 0.063
*S* = 1.04
2624 reflections
147 parameters
H-atom parameters constrained
Δρ_max_ = 0.48 e Å^−3^
Δρ_min_ = −0.70 e Å^−3^
Absolute structure: Flack (1983 ▶), 1102 Friedel pairs
Flack parameter: 0.057 (12)

### 

Data collection: *APEX2* (Bruker, 2005 ▶); cell refinement: *SAINT-Plus* (Bruker, 2004 ▶); data reduction: *SAINT-Plus*; program(s) used to solve structure: *SIR97* (Altomare *et al.*, 1999 ▶); program(s) used to refine structure: *SHELXL97* (Sheldrick, 2008 ▶); molecular graphics: *DIAMOND* (Brandenburg & Putz, 2005 ▶); software used to prepare material for publication: *WinGX* (Farrugia, 1999 ▶).

## Supplementary Material

Crystal structure: contains datablock(s) global, I. DOI: 10.1107/S1600536811044606/wn2454sup1.cif

Structure factors: contains datablock(s) I. DOI: 10.1107/S1600536811044606/wn2454Isup2.hkl

Supplementary material file. DOI: 10.1107/S1600536811044606/wn2454Isup3.cml

Additional supplementary materials:  crystallographic information; 3D view; checkCIF report

## Figures and Tables

**Table 1:** Hydrogen-bond geometry (Å, °)

*D*—H⋯*A*	*D*—H	H⋯*A*	*D*⋯*A*	*D*—H⋯*A*
N11—H11⋯O12	0.85	1.99	2.650 (4)	134
C5—H5*A*⋯Br16^i^	0.98	2.85	3.702 (4)	145
C5—H5*F*⋯Br16^i^	0.98	2.90	3.702 (4)	139
C5—H5*B*⋯Br12^ii^	0.98	2.88	3.839 (4)	168
C5—H5*D*⋯O12^iii^	0.98	2.44	3.354 (4)	155

Symmetry codes: (i) 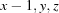; (ii) 
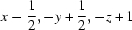
; (iii) 
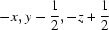
.

**Fig. 1.**
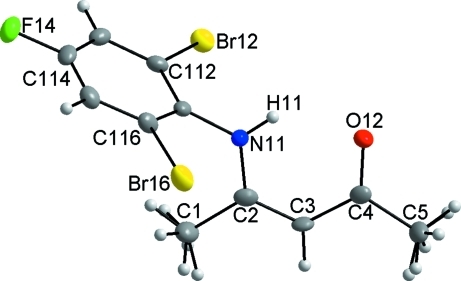
Molecular structure of the title compound. Displacement ellipsoids are drawn at the 50% probability displacement level. Hydrogen atoms are shown as spheres of arbitrary radius. Both disorder components are shown.

**Fig. 2.**
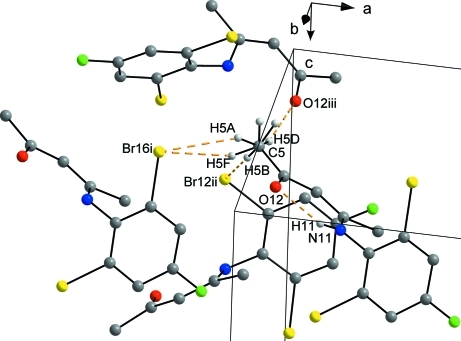
Partially filled unit cell illustrating the intra- and intermolecular hydrogen bond interactions (dashed lines) in the title compound. Hydrogen atoms not involved in these interactions have been omitted for clarity.

## supplementary crystallographic information

### Comment

A well known system in organometallic chemistry is the β-diketone compound AcacH (acetylacetone; or when coordinated acetylacetonato, acac^-^). A multitude of derivatives have been synthesized to date, with enaminoketones being one type. Enaminoketones contain nitrogen and oxygen atoms as well as an unsaturated C═C bond, making these electron-rich compounds of interest in various fields including liquid crystals (Pyżuk *et al.,* 1993), fluorescence studies (Xia *et al.,* 2008) as well as the formation of complexes of medical interest (Tan *et al.,* 2008; Chen & Rhodes, 1996). It also has significant application possibilities in catalysis (Nair *et al.,* 2002; Van Aswegen *et al.,* 1991; Steyn *et al.,* 1992, 1997; Otto *et al.,* 1998; Roodt & Steyn, 2000; Brink *et al.,* 2010). Enaminoketones readily coordinate to rhodium to form carbonyl species (Venter *et al.,* 2009*a*, 2009*b*).

The title compound (Fig. 1) is a derivative of 4-(phenylamino)pent-3-en-2-one whose crystal structure has already been published (Shaheen *et al.,* 2006) and forms part of an ongoing investigation on the influence of electron-donating and -withdrawing substituents on the benzene unit of these types of enaminoketones (Table 2; Venter *et al.,* 2010*a*, 2010*b*). The position of the substituents has an influence on the dihedral angle (angle between the benzene ring and the N—C—C—C—O plane) of the compounds, with compounds containing substituents on the *ortho*-position having larger dihedral angles. The N···O distance is larger for compounds containing electron-withdrawing substituents than for compounds containing electron-donating substituents. The C2—C3 distance of 1.355 (5) Å, *versus* the C3—C4 bond distance of 1.432 (5) Å indicates an unsaturated bond in the pentenone backbone. The dihedral angle between the benzene ring and pentenone unit is 77.2 (1)°.

Intermolecular C—H···Br and C—H···O interactions as well as an intramolecular N—H···O interaction are observed. There is also a short Br···Br contact of 3.496 (1)Å for Br12···Br16(1/2-x, 1-y, z-1/2).

### Experimental

A solution of acetylacetone (11.15 g, 0.1113 mol), 2,6-dibromo-4-fluoroaniline (26.94 g, 0.1002 mol) and 2 drops of H_2_SO_4_(conc.) in 150 ml benzene was refluxed for 24 h in a Dean-Stark trap, filtered and left to crystallize. Crystals suitable for X-ray diffraction were obtained in 30.80 g (87.59%) yield. This compound is stable in air and light over a period of several months.

### Refinement

All H atoms were placed in geometrically idealized positions and constrained to ride on their parent atoms, with C—H = 0.95 and 0.98 Å for Csp^2^—H and C(methyl)—H, respectively, N—H = 0.85 Å; *U*_iso_(H) = k*U*_eq_(carrier atom), where k = 1.2 for Csp^2^—H and 1.5 for all other H atoms. The methyl groups were positioned to fit the difference electron density and the groups were then refined as rigid rotors.  In both methyl groups each H atom is disordered equally over two sites.

### Crystal data

**Table:**

C_11_H_10_Br_2_FNO	*F*(000) = 680
*M**_r_* = 351.02	*D*_x_ = 1.93 Mg m^−^^3^
Orthorhombic, *P*2_1_2_1_2_1_	Mo *K*α radiation, λ = 0.71073 Å
Hall symbol: P 2ac 2ab	Cell parameters from 9975 reflections
*a* = 8.7710 (3) Å	θ = 2.5–26.8°
*b* = 10.8710 (4) Å	µ = 6.70 mm^−^^1^
*c* = 12.6720 (4) Å	*T* = 100 K
*V* = 1208.27 (7) Å^3^	Cuboid, colourless
*Z* = 4	0.66 × 0.25 × 0.18 mm

### Data collection

**Table:**

Bruker X8 APEXII 4K KappaCCD diffractometer	2624 independent reflections
Radiation source: fine-focus sealed tube	2381 reflections with *I* > 2σ(*I*)
graphite	*R*_int_ = 0.084
ω and φ scans	θ_max_ = 27°, θ_min_ = 2.5°
Absorption correction: multi-scan (*SADABS*; Bruker, 2004)	*h* = −11→11
*T*_min_ = 0.096, *T*_max_ = 0.379	*k* = −13→13
20084 measured reflections	*l* = −15→16

### Refinement

**Table:**

Refinement on *F*^2^	Secondary atom site location: difference Fourier map
Least-squares matrix: full	Hydrogen site location: inferred from neighbouring sites
*R*[*F*^2^ > 2σ(*F*^2^)] = 0.028	H-atom parameters constrained
*wR*(*F*^2^) = 0.063	*w* = 1/[σ^2^(*F*_o_^2^) + (0.0221*P*)^2^ + 0.0721*P*] where *P* = (*F*_o_^2^ + 2*F*_c_^2^)/3
*S* = 1.04	(Δ/σ)_max_ < 0.001
2624 reflections	Δρ_max_ = 0.48 e Å^−^^3^
147 parameters	Δρ_min_ = −0.70 e Å^−^^3^
0 restraints	Absolute structure: Flack (1983), 1102 Friedel pairs
Primary atom site location: structure-invariant direct methods	Flack parameter: 0.057 (12)

### Special details

**Table:**

Experimental. The intensity data was collected on a Bruker X8 ApexII 4 K Kappa CCD diffractometer using an exposure time of 5 s/frame. A total of 1849 frames were collected with a frame width of 0.5° covering up to θ = 26.83° with 99.9% completeness accomplished.
Geometry. All e.s.d.'s (except the e.s.d. in the dihedral angle between two l.s. planes) are estimated using the full covariance matrix. The cell e.s.d.'s are taken into account individually in the estimation of e.s.d.'s in distances, angles and torsion angles; correlations between e.s.d.'s in cell parameters are only used when they are defined by crystal symmetry. An approximate (isotropic) treatment of cell e.s.d.'s is used for estimating e.s.d.'s involving l.s. planes.

### Fractional atomic coordinates and isotropic or equivalent isotropic displacement parameters (Å^2^)

**Table:**

	*x*	*y*	*z*	*U*_iso_*/*U*_eq_	Occ. (<1)
C1	0.3941 (5)	0.1897 (4)	0.4390 (3)	0.0321 (9)	
H1A	0.4724	0.2509	0.4559	0.048*	0.5
H1B	0.4397	0.123	0.3975	0.048*	0.5
H1C	0.3517	0.1561	0.5045	0.048*	0.5
H1D	0.3702	0.1025	0.4494	0.048*	0.5
H1E	0.4028	0.2303	0.5077	0.048*	0.5
H1F	0.4908	0.1973	0.4008	0.048*	0.5
C2	0.2691 (4)	0.2495 (3)	0.3761 (3)	0.0205 (8)	
C3	0.1464 (4)	0.1850 (3)	0.3420 (3)	0.0193 (8)	
H3	0.1393	0.101	0.3621	0.023*	
C4	0.0266 (4)	0.2343 (3)	0.2780 (2)	0.0176 (8)	
C5	−0.0933 (4)	0.1488 (3)	0.2389 (3)	0.0284 (9)	
H5A	−0.1672	0.1947	0.1964	0.043*	0.5
H5B	−0.1454	0.1109	0.2991	0.043*	0.5
H5C	−0.0461	0.0846	0.1956	0.043*	0.5
H5D	−0.0719	0.0654	0.2643	0.043*	0.5
H5E	−0.0937	0.1492	0.1616	0.043*	0.5
H5F	−0.1931	0.1755	0.2651	0.043*	0.5
C111	0.4145 (4)	0.4411 (3)	0.3773 (3)	0.0177 (7)	
C112	0.4141 (4)	0.5250 (3)	0.4607 (3)	0.0183 (7)	
C113	0.5425 (4)	0.5915 (3)	0.4879 (3)	0.0213 (8)	
H113	0.5406	0.6487	0.5446	0.026*	
C114	0.6730 (4)	0.5723 (3)	0.4304 (3)	0.0232 (8)	
C115	0.6803 (4)	0.4939 (3)	0.3454 (3)	0.0222 (8)	
H115	0.7719	0.4836	0.3063	0.027*	
C116	0.5489 (4)	0.4306 (3)	0.3194 (2)	0.0202 (7)	
N11	0.2846 (3)	0.3703 (2)	0.3530 (2)	0.0198 (7)	
H11	0.212	0.4057	0.3220	0.03*	
O12	0.0192 (3)	0.3462 (2)	0.2553 (2)	0.0236 (6)	
F14	0.8008 (2)	0.6351 (2)	0.45685 (18)	0.0322 (5)	
Br12	0.23441 (4)	0.54808 (3)	0.53970 (3)	0.02556 (11)	
Br16	0.55267 (5)	0.32820 (4)	0.19895 (3)	0.03091 (12)	

### Atomic displacement parameters (Å^2^)

**Table:**

	*U*^11^	*U*^22^	*U*^33^	*U*^12^	*U*^13^	*U*^23^
C1	0.038 (2)	0.022 (2)	0.036 (2)	0.0009 (18)	−0.0122 (18)	0.0052 (18)
C2	0.029 (2)	0.0168 (17)	0.0156 (16)	0.0030 (16)	0.0038 (16)	−0.0017 (14)
C3	0.025 (2)	0.0096 (16)	0.0229 (17)	−0.0021 (16)	0.0014 (15)	0.0040 (15)
C4	0.018 (2)	0.0175 (18)	0.0176 (17)	0.0005 (15)	0.0070 (14)	−0.0024 (14)
C5	0.019 (2)	0.022 (2)	0.045 (2)	−0.0041 (16)	−0.0055 (17)	0.0049 (18)
C111	0.0196 (19)	0.0122 (16)	0.0211 (17)	0.0013 (15)	−0.0056 (14)	0.0016 (15)
C112	0.0223 (19)	0.0151 (17)	0.0176 (16)	0.0046 (14)	0.0021 (15)	0.0014 (15)
C113	0.029 (2)	0.0146 (17)	0.0205 (18)	−0.0012 (17)	−0.0043 (16)	0.0017 (14)
C114	0.0225 (19)	0.0178 (19)	0.029 (2)	−0.0069 (16)	−0.0062 (16)	0.0080 (16)
C115	0.0193 (19)	0.026 (2)	0.0210 (17)	0.0021 (16)	−0.0007 (14)	0.0065 (17)
C116	0.0238 (19)	0.0230 (18)	0.0138 (16)	0.0057 (17)	−0.0035 (15)	−0.0019 (14)
N11	0.0189 (16)	0.0144 (14)	0.0262 (15)	0.0003 (12)	−0.0063 (13)	−0.0005 (13)
O12	0.0212 (14)	0.0162 (13)	0.0333 (14)	−0.0001 (11)	−0.0054 (11)	0.0041 (12)
F14	0.0277 (12)	0.0315 (12)	0.0375 (12)	−0.0103 (10)	−0.0077 (10)	−0.0010 (11)
Br12	0.0260 (2)	0.0285 (2)	0.02217 (18)	0.00240 (17)	0.00389 (16)	−0.00038 (16)
Br16	0.0296 (2)	0.0391 (2)	0.02405 (18)	0.0117 (2)	−0.00532 (17)	−0.01234 (18)

### Geometric parameters (Å, °)

**Table:**

C1—C2	1.503 (5)	C5—H5D	0.98
C1—H1A	0.98	C5—H5E	0.98
C1—H1B	0.98	C5—H5F	0.98
C1—H1C	0.98	C111—C116	1.393 (5)
C1—H1D	0.98	C111—C112	1.397 (5)
C1—H1E	0.98	C111—N11	1.408 (4)
C1—H1F	0.98	C112—C113	1.382 (5)
C2—N11	1.353 (4)	C112—Br12	1.884 (3)
C2—C3	1.355 (5)	C113—C114	1.373 (5)
C3—C4	1.431 (5)	C113—H113	0.95
C3—H3	0.95	C114—F14	1.355 (4)
C4—O12	1.252 (4)	C114—C115	1.375 (5)
C4—C5	1.488 (5)	C115—C116	1.382 (5)
C5—H5A	0.98	C115—H115	0.95
C5—H5B	0.98	C116—Br16	1.889 (3)
C5—H5C	0.98	N11—H11	0.8453
			
C2—C1—H1A	109.5	C4—C5—H5D	109.5
C2—C1—H1B	109.5	H5A—C5—H5D	141.1
H1A—C1—H1B	109.5	H5B—C5—H5D	56.3
C2—C1—H1C	109.5	H5C—C5—H5D	56.3
H1A—C1—H1C	109.5	C4—C5—H5E	109.5
H1B—C1—H1C	109.5	H5A—C5—H5E	56.3
C2—C1—H1D	109.5	H5B—C5—H5E	141.1
H1A—C1—H1D	141.1	H5C—C5—H5E	56.3
H1B—C1—H1D	56.3	H5D—C5—H5E	109.5
H1C—C1—H1D	56.3	C4—C5—H5F	109.5
C2—C1—H1E	109.5	H5A—C5—H5F	56.3
H1A—C1—H1E	56.3	H5B—C5—H5F	56.3
H1B—C1—H1E	141.1	H5C—C5—H5F	141.1
H1C—C1—H1E	56.3	H5D—C5—H5F	109.5
H1D—C1—H1E	109.5	H5E—C5—H5F	109.5
C2—C1—H1F	109.5	C116—C111—C112	117.0 (3)
H1A—C1—H1F	56.3	C116—C111—N11	121.7 (3)
H1B—C1—H1F	56.3	C112—C111—N11	121.3 (3)
H1C—C1—H1F	141.1	C113—C112—C111	121.9 (3)
H1D—C1—H1F	109.5	C113—C112—Br12	118.6 (2)
H1E—C1—H1F	109.5	C111—C112—Br12	119.4 (2)
N11—C2—C3	120.9 (3)	C114—C113—C112	117.8 (3)
N11—C2—C1	117.4 (3)	C114—C113—H113	121.1
C3—C2—C1	121.6 (3)	C112—C113—H113	121.1
C2—C3—C4	124.7 (3)	F14—C114—C113	118.8 (3)
C2—C3—H3	117.6	F14—C114—C115	117.9 (3)
C4—C3—H3	117.6	C113—C114—C115	123.3 (3)
O12—C4—C3	122.2 (3)	C114—C115—C116	117.2 (3)
O12—C4—C5	119.6 (3)	C114—C115—H115	121.4
C3—C4—C5	118.3 (3)	C116—C115—H115	121.4
C4—C5—H5A	109.5	C115—C116—C111	122.7 (3)
C4—C5—H5B	109.5	C115—C116—Br16	118.1 (3)
H5A—C5—H5B	109.5	C111—C116—Br16	119.2 (3)
C4—C5—H5C	109.5	C2—N11—C111	124.4 (3)
H5A—C5—H5C	109.5	C2—N11—H11	117.8
H5B—C5—H5C	109.5	C111—N11—H11	117.8
			
N11—C2—C3—C4	1.8 (6)	F14—C114—C115—C116	−179.9 (3)
C1—C2—C3—C4	−177.1 (3)	C113—C114—C115—C116	−1.1 (5)
C2—C3—C4—O12	−6.2 (5)	C114—C115—C116—C111	−2.0 (5)
C2—C3—C4—C5	174.5 (3)	C114—C115—C116—Br16	177.0 (3)
C116—C111—C112—C113	−2.5 (5)	C112—C111—C116—C115	3.8 (5)
N11—C111—C112—C113	177.4 (3)	N11—C111—C116—C115	−176.2 (3)
C116—C111—C112—Br12	178.0 (2)	C112—C111—C116—Br16	−175.2 (2)
N11—C111—C112—Br12	−2.1 (4)	N11—C111—C116—Br16	4.8 (4)
C111—C112—C113—C114	−0.4 (5)	C3—C2—N11—C111	−174.1 (3)
Br12—C112—C113—C114	179.2 (3)	C1—C2—N11—C111	4.8 (5)
C112—C113—C114—F14	−178.9 (3)	C116—C111—N11—C2	75.4 (5)
C112—C113—C114—C115	2.3 (5)	C112—C111—N11—C2	−104.5 (4)

### Hydrogen-bond geometry (Å, °)

**Table:**

*D*—H···*A*	*D*—H	H···*A*	*D*···*A*	*D*—H···*A*
N11—H11···O12	0.85	1.99	2.650 (4)	134.
C5—H5A···Br16^i^	0.98	2.85	3.702 (4)	145.
C5—H5F···Br16^i^	0.98	2.90	3.702 (4)	139
C5—H5B···Br12^ii^	0.98	2.88	3.839 (4)	168.
C5—H5D···O12^iii^	0.98	2.44	3.354 (4)	155

Symmetry codes: (i) *x*−1, *y*, *z*; (ii) *x*−1/2, −*y*+1/2, −*z*+1; (iii) −*x*, *y*−1/2, −*z*+1/2.

### Table 2 Comparative geometrical parameters (Å, °) for free and coordinated N,O-bidendate (N,O-bid) compounds.

**Table:**

Parameters	I	II	III	IV
N11—C111	1.409 (4)	1.412 (3)	1.422 (2)	1.417 (2)
N11—C2	1.352 (4)	1.352 (3)	1.345 (2)	1.348 (1)
O12—C4	1.252 (4)	1.244 (3)	1.257 (2)	1.253 (1)
C2—C3	1.355 (5)	1.365 (4)	1.383 (3)	1.384 (2)
C3—C4	1.432 (5)	1.424 (4)	1.420 (2)	1.424 (2)
N11···O12	2.650 (4)	2.658 (3)	2.635 (2)	2.646 (1)
N11—C2···C4—O12	-3.8 (3)	1.4 (2)	-0.5 (1)	1.70 (9)
Dihedral angle	77.2 (1)	32.03 (9)	49.53 (5)	29.90 (3)

(I) This work; (II) 4-(phenylamino)pent-3-en-2-onato [Shaheen *et al.* (2006)]; (III) 4-(2-methylphenylamino)pent-3-en-2-onato [Venter *et al.* (2010*a*)]; (IV) 4-(4-methylphenylamino)pent-3-en-2-onato [Venter *et al.* (2010*b*)]. The dihedral angle is defined as the torsion angle between the N—C—C—C—O plane and the benzene ring. A positive angle denotes a clockwise rotation.
